# Identification of proteins that interact with the UL24 protein of herpes simplex virus 1

**Published:** 2011-01-10

**Authors:** L Bertrand, A Pearson

**Affiliations:** 1 INRS-Institut Armand Frappier, 531, Blvd. Des Prairies, Laval, Quebec, Canada

## 

Infection with herpes simplex virus 1 (HSV-1) typically results in the formation of cold sores; however, in individuals with a compromised immune system the infection can be severe. The virus life cycle is characterized by two distinct phases: a lytic replication phase occurring in mucous membranes, and a latent state that is established in neurons of the trigeminal ganglia.

The UL24 protein of HSV-1 is implicated in both the lytic and latent phases of infection. Infection with a UL24-deficient virus *in vitro* results in reduced viral yields and the formation of syncytial plaques, where the plasma membranes of infected cells fuse together. *In vivo*, the absence of UL24 results in reduced viral titers and a drastic reduction in viral reactivation *ex vivo*. Previously, we showed that UL24 is both necessary and sufficient to induce the redistribution of the nucleolar protein nucleolin. The UL24 protein contains 269 amino acids and is expressed late in the virus life cycle. A bioinformatics study identified an endonuclease motif within the conserved N-terminal portion of the protein; however, its function remains to be demonstrated. Although several roles have been attributed to UL24, the mechanisms involved are unknown.

We hypothesized that UL24 functions, at least in part, through protein-protein interactions. To investigate this possibility, we used a virus that expresses UL24 with a hemagglutinin (HA) tag fused to its N-terminus, vHA-UL24. Lysates from infected cells were harvested, and fractionated on glycerol gradients. We found that UL24 was present in fractions corresponding to a much higher molecular mass than would be expected for UL24 alone, suggesting an association of UL24 with cellular or viral proteins during infection. To isolate potential UL24-binding proteins, we performed immunoprecipitations on fractions containing HA-UL24, using antibodies directed against the HA epitope. Immunoprecipitated proteins were resolved by denaturing polyacrylamide gel electrophoresis. Silver staining of the gel enabled the detection of proteins that appeared to specifically co-precipitate with HA-UL24. Candidate binding proteins will be identified by mass spectrometry. The identification of UL24-interacting partners will contribute to a better understanding of the roles of UL24 in different steps of the virus life cycle.

